# Vitamin C is a source of oxoaldehyde and glycative stress in age‐related cataract and neurodegenerative diseases

**DOI:** 10.1111/acel.13176

**Published:** 2020-06-21

**Authors:** Xingjun Fan, David R. Sell, Caili Hao, Sabrina Liu, Benlian Wang, Daniel W. Wesson, Sandra Siedlak, Xiongwei Zhu, Terrance J. Kavanagh, Fiona E. Harrison, Vincent M. Monnier

**Affiliations:** ^1^ Department of Cellular Biology and Anatomy Medical College of Georgia at Augusta University Augusta Georgia; ^2^ Department of Pathology Case Western Reserve University Cleveland Ohio USA; ^3^ Lakeside High School Augusta Georgia; ^4^ Center for Proteomics Case Western Reserve University Cleveland Ohio USA; ^5^ Neurosciences Case Western Reserve University Cleveland Ohio USA; ^6^ Department of Environmental and Occupational Health Sciences University of Washington Seattle Washington USA; ^7^ Department of Medicine Vanderbilt University Medical Center Nashville Tennessee USA; ^8^ Biochemistry Case Western Reserve University Cleveland Ohio USA; ^9^ Present address: Department of Pharmacology & Therapeutics University of Florida Gainesville Florida USA

**Keywords:** Alzheimer's disease, catalytic metals, glycation, lens, methylglyoxal, Parkinson's disease

## Abstract

Oxoaldehyde stress has recently emerged as a major source of tissue damage in aging and age‐related diseases. The prevailing mechanism involves methylglyoxal production during glycolysis and modification of arginine residues through the formation of methylglyoxal hydroimidazolones (MG‐H1). We now tested the hypothesis that oxidation of vitamin C (ascorbic acid or ASA) contributes to this damage when the homeostatic redox balance is disrupted especially in ASA‐rich tissues such as the eye lens and brain. MG‐H1 measured by liquid chromatography mass spectrometry is several fold increased in the lens and brain from transgenic mice expressing human vitamin C transporter 2 (hSVCT2). Similarly, MG‐H1 levels are increased two‐ to fourfold in hippocampus extracts from individuals with Alzheimer's disease (AD), and significantly higher levels are present in sarkosyl‐insoluble tissue fractions from AD brain proteins than in the soluble fractions. Moreover, immunostaining with antibodies against methylglyoxal hydroimidazolones reveals similar increase in substantia nigra neurons from individuals with Parkinson's disease. Results from an in vitro incubation experiment suggest that accumulated catalytic metal ions in the hippocampus during aging could readily accelerate ASA oxidation and such acceleration was significantly enhanced in AD. Modeling studies and intraventricular injection of ^13^C‐labeled ASA revealed that ASA backbone carbons 4–6 are incorporated into MG‐H1 both in vitro and in vivo, likely via a glyceraldehyde precursor. We propose that drugs that prevent oxoaldehyde stress or excessive ASA oxidation may protect against age‐related cataract and neurodegenerative diseases.

## Graphical abstract

Oxoaldehyde stress in aging and age‐related diseases is widely believed to stem from advanced glycation end products of glucose via methylglyoxal formation during glycolysis. Here, we demonstrate that ascorbic acid oxidation is a source of damage via methylglyoxal hydroimidazolone formation in long‐lived proteins from tissues rich in vitamin C such as the lens and the brain.

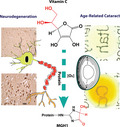

## 1 INTRODUCTION

Ascorbic acid (vitamin C, ASA) is a key component of the redox system that protects the cell from oxidant stress in conjunction with glutathione and other redox‐active molecules. Low dietary intake contributes, along with other disorders, to cataractogenesis and neuronal disorders in experimental animals and humans (Tan et al., 2008). Strong experimental data in the animal suggest that depleted ASA levels contribute to cognitive impairment in Alzheimer's disease (Harrison, May, & McDonald, 2010). Yet, ASA itself can be a source of advanced glycation end products (AGEs) raising the paradoxical question of whether the vitamin can have both anti‐aging and pro‐aging properties.

AGEs are chemical structures nonenzymatically formed from the Maillard reaction between reducing sugars such as glucose, with amino groups in proteins, lipids, and nucleic acids. Levels of most AGEs increase with age in tissues with slow protein turnover, such as those of the lens and brain (Ahmed et al., 2003; Horie et al., 1997). Notable examples include *N^ε^*‐carboxymethyl‐lysine (CML), pentosidine, and glucosepane. Among AGEs, the methylglyoxal hydroimidazolone of arginine, MG‐H1, is among the most prevalent cellular AGE, corresponding to about 1%–2% of total arginine residues of proteins in mammalian tissue, plasma, and extracellular matrix proteins (Rabbani & Thornalley, 2012). In aged human lenses, levels of MG‐H1 up to 14 nmol/mg protein were reported (Ahmed et al., 2003). Our own comparative studies confirmed that MG‐H1 is the most abundant AGEs, with levels reaching 6 nmol/mg protein in aged human lenses (Fan et al., 2010). Such levels may significantly affect crystallin conformation and chaperone function (Mukhopadhyay, Kar, & Das, 2010). Outside the lens, MG‐H1 is a ligand for the RAGE receptor whose engagement triggers a pro‐inflammatory cascade (Litwinoff, Hurtado Del Pozo, Ramasamy, & Schmidt, 2015).

Methylglyoxal (MGO), a reactive tricarbon α‐oxoaldehyde, is considered the major precursor for MG‐H1. The biochemical origin of cellular MGO is widely attributed to anaerobic glycolysis, via fragmentation of triose phosphate and to a smaller extent to catabolism of ketone bodies and threonine (Phillips & Thornalley, 1993). Because of its toxicity as a highly reactive glycation agent, MGO levels in cultured cells, plasma, and animal tissue are in the picomolar range to low nanomolar range (Rabbani & Thornalley, 2014) whereby glyoxylase I and glyoxylase II are the primary detoxification systems with the help of glutathione (Rabbani & Thornalley, 2012).

In the work below, we propose that ASA can be an important source of MG‐H1‐mediated protein damage in ASA‐rich tissues such as the lens and the brain. Growing evidence has shown that ASA contributes to nonenzymatic modification of proteins in the old and cataractous lens (Fan et al., 2006). Concentrations in these tissues can reach 3‐5 mM. This hypothesis is further supported by a mouse model in which we overexpressed the human sodium‐dependent vitamin C transporter 2 (hSVCT2) in the lens, thereby increasing the influx of ASA as well as total lens browning and AGE accumulation (Fan et al., 2006). The chemical pathways by which ascorbic acid generates glycating agents implicate its oxidized form dehydroascorbic acid (DHA). In vivo, DHA can be either reduced back to ASA via the ascorbate–glutathione cycle or further broken down via an irreversible molecular pathway. The major DHA degradation products have been identified in recent years (Smuda & Glomb, 2013). These include 2,3‐diketogulonic acid (2,3‐DKG), L‐erythrulose, oxalate, L‐threo‐pentos‐2‐ulose (L‐xylosone), 3,4‐dihydroxy‐2‐oxobutanal (L‐threosone), and glyceraldehyde (Smuda & Glomb, 2013). Several AGEs were identified as a result of ascorbic acid or DHA reaction with proteins, such as CML, pentosidine, and vesperlysine A (Dunn et al., 1990).

Below, we have carried out a systematic analysis of ascorbic acid degradation in vitro and its role in MG‐H1 formation in hSVCT2 transgenic mouse lens and brain tissue using a combination of immunohistochemistry and mass spectrometry with isotopically labeled ^13^C‐ascorbic acid. We also present data with human tissues from individuals with Alzheimer's disease (AD) and Parkinson's disease (PD) and show that metal‐catalyzed ascorbate oxidation is involved in MG‐H1 formation.

## 2 RESULTS

### 2.1 Ascorbic acid oxidation and ascorbylation produce MG‐H1 from in vitro incubation and hSVCT2 transgenic mouse

**FIGURE 1:**
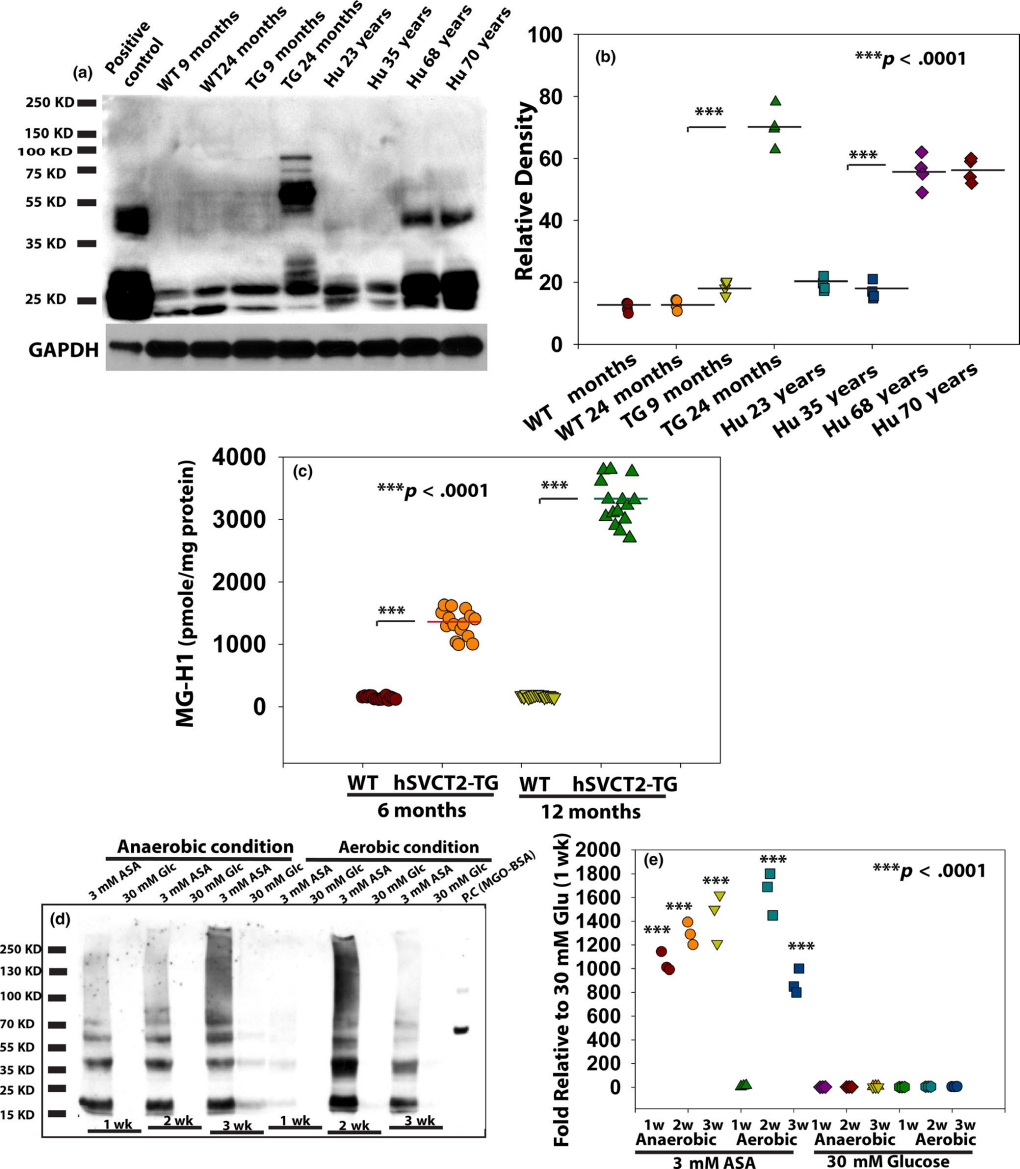
MG‐H1 was detected in aged human lens, hSVCT2 transgenic mouse lens, and in vitro incubated lens protein extract with ascorbic acid (ASA). ASA carbons C4, C5, and C6 are involved in MG‐H1 formation based on ^13^C‐ASA labeling proteomics. (a) MG‐H1 formation detected in wild‐type mouse (WT) and in hSVCT2 transgenic (TG) mouse lens extract at 9 and 24 months, and in human lenses at 23, 35, 68, and 70 years old, respectively, by Western blot using MG‐H1 antibody. Lens protein extract incubated with MGO was used as positive control, and GAPDH was used as loading control. (b) Semi‐quantitative analysis of the MG‐H1 levels from (a). (c) MG‐H1 levels determined by LC/MS analysis in a separate experiment indicate a significant increase in hSVCT2 TG mice lens extract compared with WT at both 6 months and 12 months old (*n* = 15/group, *p* < .0001). (d) Incubation of 4‐month‐old human lens protein extract with ASA (3 mM), but not glucose (Glc, 30 mM), at 37°C for 1–3 weeks produced high levels of MG‐H1 in both aerobic and anaerobic conditions. Bovine serum albumin (BSA) incubated with 100 µM MGO for 7 days served as a positive control. (e) Semi‐quantitative analysis of the MG‐H1 levels from (d)

The first and strongest in vivo evidence for the role of ASA in MG‐H1 formation comes from the analysis of our lens‐specific hSVCT2 transgenic mouse study. MG‐H1 presence was detected in mouse lens protein extract using immunoblot, as shown in Figure 1a and b. A weak MG‐H1 signal could be detected in WT mice starting at 9 months that was stronger in lenses from hSVCT2 mice. A very strong signal was detected at 24 months that mimicked the one found in 68 and 70‐year‐old human lens‐soluble protein (Figure 1a and b). In separate studies, LC/MS analysis confirmed elevated MG‐H1 levels in hSVCT2 transgenic mouse lenses at both 6 and 12 months of age (*p* < .0001) (Figure 1c). We initially attributed this finding to elevated free methylglyoxal (MGO) in hSVCT2 transgenic lens. However, attempts to measure the free MGO level in these transgenic mice using the o‐phenylene diamine derivatization method failed to reveal changes in MGO levels. Mean detected levels were 5.45 ± 0.63 nmole/g wet weight and 5.92 ± 0.57 nmole/g wet weight in WT and hSVT2 mice (p = NS), respectively.

The inability to detect increased levels of free MGO in hSVCT2 lens led us to investigate the mechanistic relationship between ASA oxidation and MG‐H1 formation. Four‐month‐old human lens protein extract was incubated at 37°C for one to three weeks with either 3 mM ASA or 30 mM glucose in both anaerobic and aerobic conditions at 37°C. As illustrated in Figure 1d and e, ASA oxidation and ascorbylation led to the formation of easily detectable levels of MG‐H1 during 1 week to 3 weeks as detected by immunoblot analysis. In contrast, 30 mM glucose produced barely detectable MG‐H1 in either anaerobic or aerobic incubation conditions, even after a 3‐week incubation. Since aerobic conditions did not consistently enhance MG‐H1 formation compared with anaerobic conditions following incubation with ASA, this suggests that the “nonoxidative dehydroascorbic acid catabolism pathway” (Simpson & Ortwerth, 2000) might produce the precursor responsible for MG‐H1 formation. We did notice a reduced immunoblot signal at 3‐week incubation under aerobic condition with 3 mM ASA that we believe is due to precipitation (Ortwerth & Olesen, 1988). Indeed, in similar studies in progress we observed a 34% loss of MG‐H1‐rich ascorbylated proteins into the pellet.

### 2.2 Ascorbic acid backbone carbons 4, 5, and 6 are part of MG‐H1 structure

**FIGURE 2:**
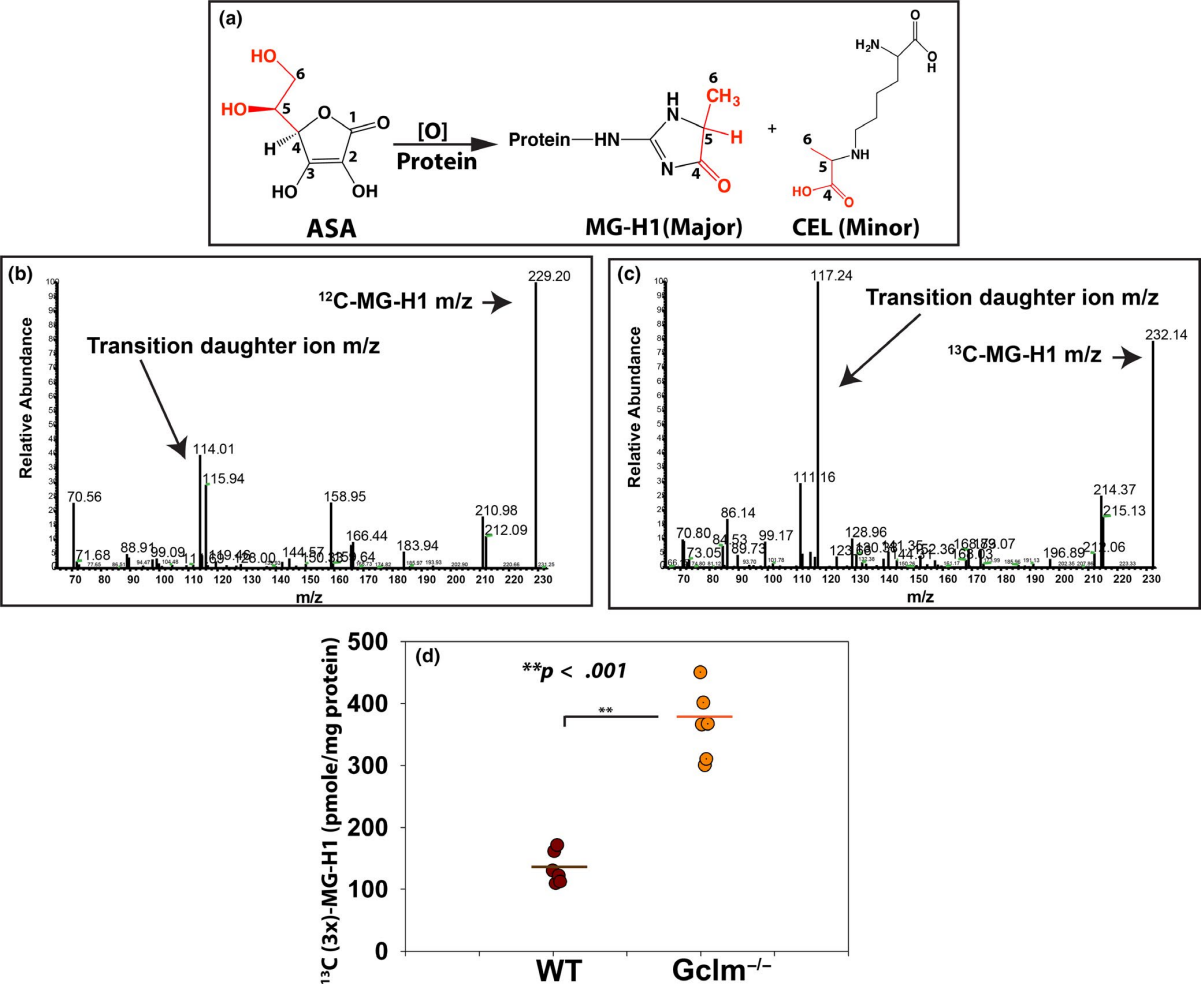
In vitro modification by ^13^C‐labeled ASA at carbon positions 1 and 2, 3, or 5 implicated that the C4‐6 backbone of ASA was incorporated into MGH1 and CEL structures as illustrated in (a), and in vivo direct evidence of MG‐H1 formation by bilateral brain ventricles U_6_‐^13^C‐ASA injection. (b) MG‐H1 mass (m/z 229) and transition fragment ion (m/z 114) were determined in control mice brain tissue without ASA injection. (c) ^13^C (3x)‐MG‐H1 mass (m/z 232) and fragment ion (m/z 117) were detected in both WT and Gclm^−/−^ mice brain tissue 2 weeks after ASA injection. (d) ^13^C (3x)‐MG‐H1 was detected in both WT and Gclm^−/−^ mice brains, but significantly higher levels (*p* < .001) of ^13^C (3x)‐MG‐H1 were found in the brains of Gclm^−/−^ mice compared with those of WT mice

To clarify the mechanism of MG‐H1 formation in the presence of ascorbic acid, we used ^13^C‐ascorbate labeled at three different carbon positions, that is, 1 and 2, or 3 or 5, to modify 4‐month‐old human lens protein extract and detect the protein‐bound MG‐H1 using mass spectrometry and proteomic analysis. Table 1 lists all tryptic peptides from human alpha A crystallin (CRYAA) of the lens protein extract that were found modified at lysine or arginine residues upon incubation with either of the ^13^C‐ASA species. The mass spectra are displayed in Figs S1a‐d (also in Table 1). For C_1_ and C_2_ and C_3_^13^C‐labeled ASA, 100% of MG‐H1 identified by mass spectra was constituted by ^12^C carbons. Similarly, N^ε^‐carboxyethyl‐lysine (CEL), another AGEs that can be derived from MGO, was also uniquely composed of ^12^C carbons. In contrast, 100% of ^13^C‐ MG‐H1 was constituted from ^13^C carbons derived from ^13^C_5_‐ASA incubations. Also, 100% detected ^13^C‐CEL was composed of ^13^C_5_ carbons. No MG‐H1 was detected in 4‐month‐old human lens extract without ASA modification. These results indicate that ASA underwent C_3_–C_4_ break during degradation with subsequent C_4‐6_ backbone incorporation into MG‐H1 or CEL structure (Figure 2a).

**TABLE 1:** Identified alpha A crystallin (CRYAA) peptides with MG‐H1 or CEL modification arising from incubation with ascorbic acid with ^13^C labeled at different positions

Residue number	Peptide	Mod. Site	Peptide m/z	Error (ppm)	Modification detected	Mass shift (Da)	Detection of ^13^C‐label depending on position in ASA		
							C_13_ (1,2)	C_13_ (3)	C_13_ (5)
12–21	**R**TLGPFYPSR	**R12**	**624.8354 (2+)**	**−2**	**MG‐H1(^13^C)**	**55.0139**			**X(39)**
50–65	QSLF**R**TVLDSGISEVR	R60	930.9935 (2+)	−1	MG‐H1	54.0106	X^a^ (26)^b^	X (27)	
66–78	SDRD**K**FVIFLDVK	K70	551.9650 (3+)	−2	CEL	72.0211	X (37)	X (42)	
		**K70**	**827.9526 (2+)**	**0**	**CEL(^13^C)**	**73.0245**			**X (55)**
100–112	HNE**R**QDDHGYISR	R103	560.9194 (3+)	−2	MG‐H1	54.0106	X (33)	X (33)	
		**R103**	**561.2579 (3+)**	**−1**	**MG‐H1(^13^C)**	**55.0139**			**X (26)**
146–163	IQTGLDATHAE**R**AIPVSR	R157	663.6875 (3+)	−1	MG‐H1	54.0106	X (25)	X (28)	

^a^ X indicates that the modification was detected.

^b^ Mascot score.

Bold format is used to highlight all modifications that are due to carbon 5 and not carbons 1‐3 from ^13^C‐labeled ascorbic acid.

### 2.3 Direct evidence for in vivo MG‐H1 formation from ASA Oxidation and Ascorbylation

Our in vitro incubation results provide direct evidence for a role of ASA in MG‐H1 formation, but the evidence from our lens‐specific hSVCT2 transgenic mouse is indirect. In order to prove that ASA oxidation is involved in MG‐H1 formation in vivo, we performed stereotaxic injection at each lateral ventricle with 10 μl of 20 mM universally ^13^C‐labeled ASA (U6‐^13^C‐ASA) in both wild‐type and Gclm knockout mouse (Gclm^−/−^). Gclm mice lack the noncatalytic enzyme subunit of γ‐glutamyl cysteine ligase. Thus, compared to WT, the Gclm^−/−^ mouse has around 60% less reduced glutathione (GSH) levels, which is mainly responsible for maintaining the ascorbic acid in its reduced form in vivo. Two weeks after injection, mouse brain was dissected and processed by acid hydrolysis for the determination of native ^12^C‐MG‐H1 and injected, ascorbate‐derived, ^13^C(3x)‐MG‐H1 by LC/MS using multiple reaction monitoring (MRM) program to simultaneously detect both the molecular ion m/z 229 and transition daughter ion *m/z* 114 for ^12^C‐MG‐H1, and m/z 232 and transition daughter ion m/z 117 for ^13^C(3x)‐MG‐H1, respectively (Figure 2b–c). Two of six Gclm KO but no control mice died from a seizure after injection, though the reason is unknown. Both ^12^C‐MG‐H1 and ^13^C(3x)‐MG‐H1 (m/z + 3) were quantitatively determined by LC/MS after acid hydrolysis. As expected, ^13^C(3x)‐MG‐H1 was present in both WT and Gclm KO mouse brain protein hydrolysates whereby a twofold increase in mean levels of injected ascorbate‐derived, ^13^C(3x)‐MG‐H1 was found in Gclm KO mice compared with WT mouse (*p* < .001, Figure 2d).

### 2.4 The rate of MG‐H1 formation from ASA and its catabolic compounds versus methylglyoxal

**FIGURE 3:**
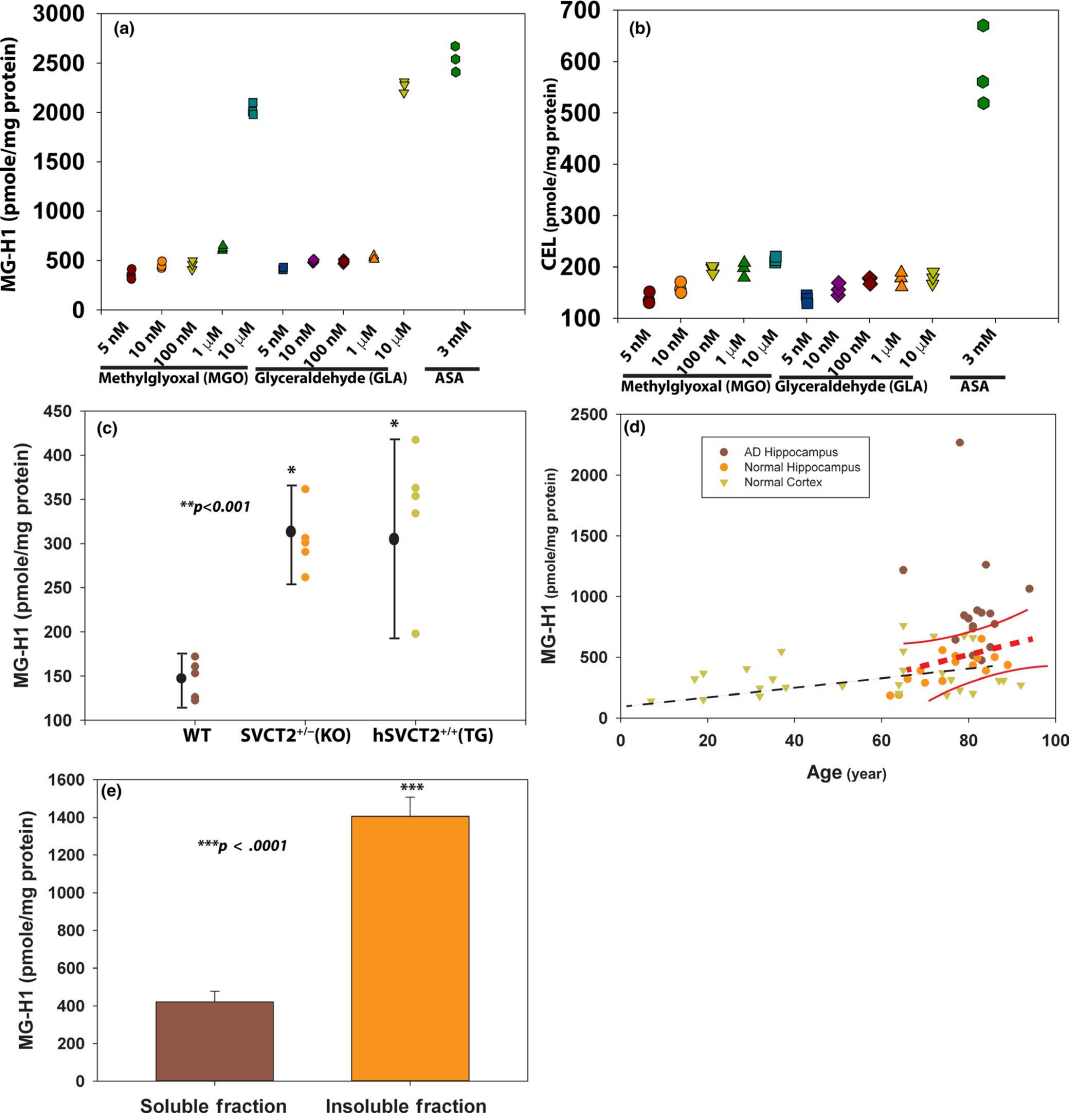
In vitro modeling of MG‐H1 formation as a function of candidate metabolic precursors and MG‐H1 determination from mouse and human brain tissue. Physiological concentration of ASA produced much higher levels of MG‐H1 and CEL than MGO at supraphysiological concentrations (<4 μM). 20 mg/ml of BSA was incubated with 3 mM ASA or various concentrations of MGO or glyceraldehyde at 37°C for one week in anaerobic condition. The MG‐H1 and CEL were analyzed by LC/MS after protease digestion. (a) MG‐H1. (b) CEL. Triplicates were used in each data point. MG‐H1 levels determined by LC/MS after protease digestion in systemic SVCT2 transgenic mouse brain cortex (*n* = 5) and in human brain comprising nondiseased human brain cortex (*n* = 30), AD hippocampus (*n* = 16), age‐matched normal hippocampal brain (*n* = 15), and AD hippocampal‐soluble and sarkosyl‐insoluble fractions (*n* = 8). (c) Brain cortex of 9‐month‐old systemic SVCT2 transgenic mice and SVCT2^±^ heterozygous knockout mice showed elevated MG‐H1 formation compared with WT mice (*p* < .001). (d) Human normal brain cortex (filled inverted triangle) MG‐H1 increased with age. The regression line is shown (*y* = 249.31 + 1.87*x*, *r* = 0.66, *p* < .0001, *n* = 30). The AD hippocampal (filled brown circle) and age‐matched normal hippocampal ( filled yellow circle) MG‐H1 levels are also shown in the same graph. Greater MG‐H1 levels were also recorded for AD hippocampal samples compared with normal controls. The regression line and 95% confidence intervals are shown in red color (*y* = 903.62 + 0.079*x*, *r* = 0.49, *p* = .002, *n* = 16). (e) MG‐H1 levels in sarkosyl‐insoluble fractions of AD hippocampus were greater than threefold increase compared with soluble fractions (*p* < .0001)

The above data provide strong evidence that oxidized ascorbic acid can attack, both in vivo and in vitro, the guanidino group of protein arginine residues to form MG‐H1 from carbons C_4_–C_6_. While this work was in progress, Smuda and Glomb showed that glyceraldehyde (GLA) was a major decomposition product of ASA under anaerobic conditions, also originating from C_3_‐C_6_, while MGO levels were minimal (Smuda & Glomb, 2013). This raised the question of how lenticular ASA concentrations could account for the observed modifications. While the MGO level in the aged human lens (46–78 year) is higher than in plasma, though still less than 2 μM (Rabbani & Thornalley, 2014), human lens and brain ASA levels are up to 100‐ and 200‐fold higher than in plasma (50 µM), respectively. In order to study the role ascorbic acid plays in MG‐H1 formation in the aged human lens, we quantitatively compared the formation of MG‐H1 from MGO versus glyceraldehyde and ASA at an expected physiological concentration of ascorbic acid. We incubated these compounds with bovine serum albumin (BSA) in anaerobic condition at 37°C for 7 days. The MG‐H1 formation was subsequently determined by LC/MS analysis. As shown in Figure 3a, both MGO and glyceraldehyde produced similar levels of MG‐H1 at identical concentrations. Unexpectedly, 3 mM ascorbic acid produced around 2.2 μmole MG‐H1 in every milligram protein, which is equivalent to 10 μM of MGO or glyceraldehyde. Interestingly, 3 mM ascorbic acid produced threefold more CEL than 10 μM MGO and glyceraldehyde (Figure 3b). These results suggest that, at physiological concentrations, ascorbic acid via glyceraldehyde is the major MG‐H1 precursor in human lens MG‐H1 formation (Figure 3a).

### 2.5 Protein‐bound MG‐H1 is significantly elevated in the brain of systemic SVCT2 transgenic mice and is highly associated with human brain aging, Alzheimer's disease, and Parkinson's disease

Besides leukocytes and the adrenal gland, the brain is the only other organ to maintain high ASA levels as in the lens. Some studies suggest that neuronal levels reach as much as 10 mM levels (Harrison & May, 2009). For this reason, we tested by LC/MS the in vivo MG‐H1 formation in mouse brain from 9‐month‐old homozygous SVCT2 transgenic mouse expressing the vitamin C transporter 2 systemically. There was a twofold elevation of MG‐H1 in transgenic versus age‐matched WT mouse brain (Figure 3c), suggesting that 9‐month‐old transgenic mice are unable to keep the excess ASA entirely in its reduced form. Surprisingly and importantly, we found the same pattern of increased MG‐H1 in SVCT2^±^ heterozygous mice in spite of a previously reported 50% reduction in expression of the SVCT2 and 30% less ASA (Dixit et al., 2015). This suggests that low cellular ASA levels foster MG‐H1 formation either due to accumulation of DHA linked to elevated oxidative stress or via ROS production and inactivation of glyceraldehyde phosphate dehydrogenase. Such alternative mechanism of MG‐H1 production in low ASA environment remains to be investigated.

Given that the same chemical changes appear to occur in brain and lens in mice, in ASA deficient as well as artificially elevated ASA conditions, these findings may also be applicable to an aged human brain as well as Alzheimer's disease (AD) and Parkinson's disease (PD) brain. A positive association of MG‐H1 in cerebrospinal fluid (CSF) (Ahmed et al., 2005) with AD has been reported, but, to our knowledge, no data are available on protein‐bound MG‐H1 in aged normal as well as AD human brain tissue. We prepared human brain protein extracts from 30 normal brain cortex and 16 AD brain hippocampus and 15 age‐matched healthy hippocampus tissues. Protein‐bound MG‐H1 by mass spectrometry significantly correlated with age (*r* = 0.66, *p* < .0001) in normal brain frontal cortex (Figure 3d). Second, AD brain hippocampus showed significant higher (*p* = .002) level of MG‐H1 relative to the age‐matched healthy brain (Figure 3d). Further separation of the hippocampus and frontal cortex samples (*n* = 8) into an insoluble fraction with the use of sarkosyl, a SDS‐like detergent used to extract insoluble aggregates, showed MG‐H1 level in AD‐insoluble fractions was quite significantly higher than in AD‐soluble fractions (*p* < .0001) (Figure 3e).

**FIGURE 4:**
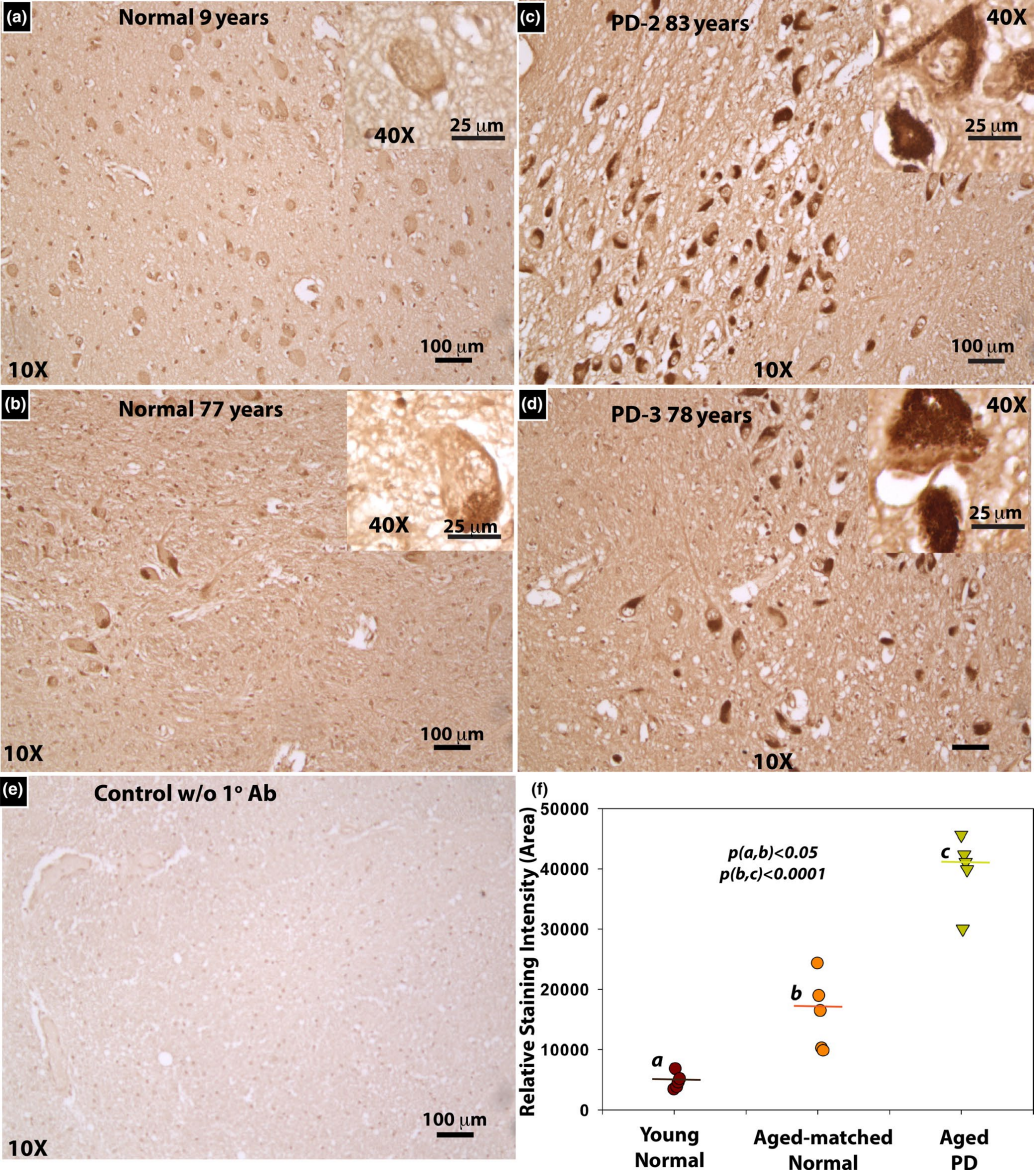
Immunolocalization of MG‐H1 reveals intense histochemical presence of MG‐H1 in PD substantia nigra compared with young and age‐matched control midbrain. Representative images of MG‐H1 in substantia nigra region of (a) 9‐year‐old and (b) 77‐year‐old normal brains; (c) 83‐year‐old and (d) 78‐year‐old PD brains are shown. The main images are taken at 10x magnification and the insets in upper right are 40x. (e) Stain without primary antibody on 83‐year‐old PD brain. (f) Statistical comparison of staining intensity between young normal, aged normal, and PD substantia nigra based on ImageJ semi‐quantitative analysis

Given the fact that GSH levels are decreased over 40% in the substantia nigra from Parkinson's disease (PD) patients compared with controls subjects (Sian et al., 1994), we surveyed the localization of MG‐H1 using immunohistochemical staining with a monoclonal MG‐H1 antibody. Profound MG‐H1 immunoreactivity was found in neurons in the substantia nigra in human PD subjects, as illustrated in Figure 4. We also saw more intense staining of MG‐H1 in the substantia nigra in aged healthy compared with young control subjects (Figure 4a–b) but much less than PD subjects (Figure 4c–d also in Figure S2). Control immunostaining without primary but only secondary enzyme‐linked antibody was negative (Figure 4e). Semi‐quantitative analysis using ImageJ (Figure 4f) confirmed these findings.

### 2.6 Mechanistic studies implicated metal ion accumulation in aging and Alzheimer's disease (AD) facilitates ASA oxidation and MG‐H1 formation

**FIGURE 5:**
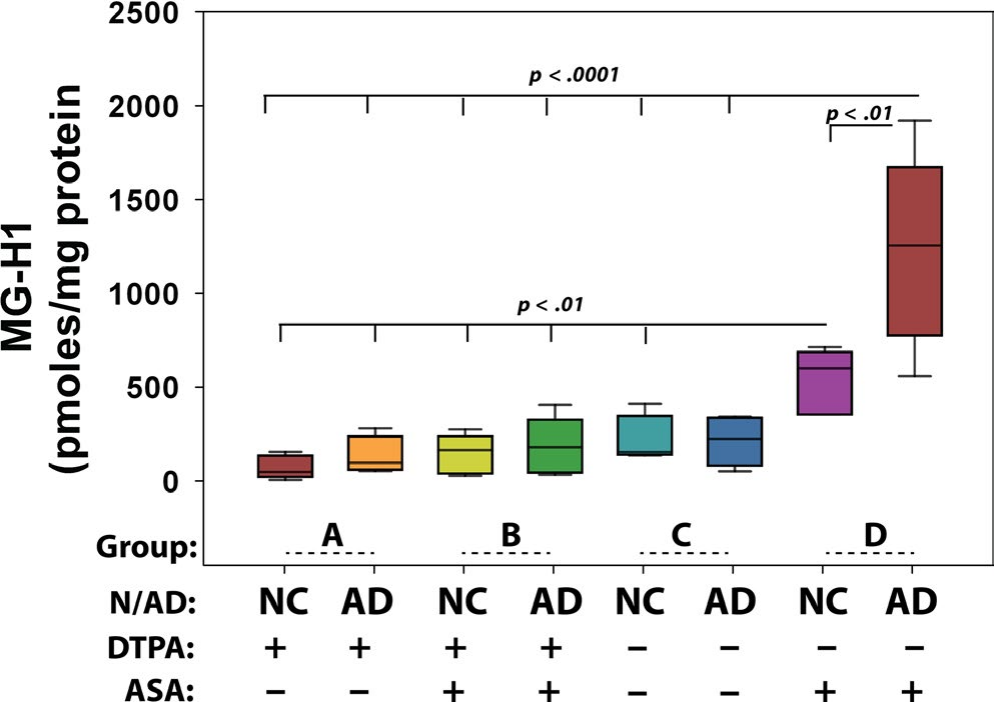
Tissue‐bound catalytic metal ions promote ASA oxidation and MG‐H1 formation. Hippocampal tissues from AD and age‐matched control (*n* = 5) were processed in four conditions. Group A and group B tissue homogenates were dialyzed in buffer containing 5 mM DTPA to deplete metal ions from buffer and tissue, and group C and group D tissues were directly homogenized in metal‐free Chelex‐treated sodium phosphate buffer to prevent buffer mediate ASA oxidation but preserve native tissue‐bound catalytic activity. 3 mM final concentration of ASA was included in group B and group D samples. All groups were incubated at 37°C for 48 hr, and MG‐H1 was analyzed by LC/MS and expressed as pmol per milligram protein. NC, normal healthy control; AD, Alzheimer's disease. One‐way ANOVA was used to compare between groups, and only *p* < .05 is considered significant

ASA oxidation is considerably accelerated in the presence of catalytic metal ions, such as iron and copper. Growing evidence suggests that there is an age‐related increase in essential and nonessential metal ions levels in the human brain, and much higher levels are found in selected brain regions in neurodegenerative diseases (Ramos et al., 2014). To test whether the catalytic metal ions in aged brain tissue can enhance ASA oxidation and MG‐H1 formation, we performed in *vitro* incubations of human AD and healthy control hippocampal tissue protein extract with or without ASA under two conditions. Some tissues were homogenized and dialyzed to deplete catalytic metal ions using the chelator diethylenetriamine pentaacetic acid (DTPA), a strong chelating reagent (Figure 5, groups A and B). Other tissues were homogenized directly in the same phosphate buffer in which catalytic metals were first removed by precipitation using the resin Chelex (Figure 5, groups C and D). Clearly, DTPA‐mediated stripping of tissue‐bound metals significantly suppressed ASA oxidation and MG‐H1 formation by protein extract from AD hippocampus, while the latter dramatically enhanced ASA oxidation compared with age‐matched healthy control. Importantly, the outcome of these experiments should alleviate any concern of contamination of commercial batches of ASA, as no MG‐H1 formed in DTPA‐incubated samples (group B data).

## 3 DISCUSSION

Oxoaldehyde stress is recognized as a threat to health in various metabolic conditions and diseases of accelerated aging. While it is commonly attributed to leakage of methylglyoxal during glycolysis or Maillard‐catalyzed autoxidation of reducing sugars as a minor source, the above study points to the paradigm‐shifting observation that oxoaldehyde stress might originate from ascorbic acid oxidation rather than glucose in ascorbic acid‐rich cells as in neurons and the lens fiber cells.

In diabetes, for example, increased serum MG‐H1 levels are known to correlate highly with diabetic retinopathy in both type I and type II diabetes mellitus (TDM) (Fosmark et al., 2009). Similarly, MG‐H1 formation in skin collagen from diabetic participants in the DCCT/EDIC study revealed highly significant associations with future progression of retinopathy and neuropathy (Genuth et al., 2015). MG‐H1 correlates with the future carotid intima thickness (Monnier, Genuth, & Sell, 2016), and MG‐H1 modification of LDL is linked to increased atherogenicity and cardiovascular disease risk (Rabbani et al., 2011). In neurodegenerative diseases, both protein‐bound and protein‐free MG‐H1 levels in cerebrospinal fluid (CSF) are associated with Alzheimer's disease and linked to cognitive impairment (Ahmed et al., 2005).

Various mechanisms have been put forth to explain the toxicity of oxoaldehyde stress, and the reader is referred to recent comprehensive review by Rabbani & Thornalley (2015). One mechanism is thought to involve the modification of reactive amines in key proteins, lipids, or DNA (Waris et al., 2015) and the inactivation of key enzymes and denaturation of structural proteins (Pietkiewicz, Bronowicka‐Szydelko, Dzierzba, Danielewicz, & Gamian, 2011). MG‐H1 and carboxyethyl‐lysine (CEL) modifications in proteins can also act as ligands for the engagement RAGE and pro‐inflammatory pathways that are known to participate in many diseases of aging (Kuhla, Ludwig, Kuhla, Munch, & Vollmar, 2015). Direct proof of methylglyoxal toxicity was brought forth in 2014 by Vlassara and colleagues who found that mice fed with MG‐H1 diet for 18 mos developed dementia and metabolic syndrome (Cai et al., 2014).

Driven by active transport, vitamin C levels are unusually high in tissues such as lens, brain, and adrenal gland, whereby its degradation products inflict substantial protein damage via AGE formation. However, the pathways of in vivo vitamin C degradation are still poorly understood because differentiating its catabolites from other endogenous sources of carbohydrates and reactive carbonyl compounds is not possible without tracers. Thus, understanding the contribution of vitamin C oxidation products to protein modification by the Maillard reaction is intrinsically difficult. In that regard, the above studies now provide unequivocal evidence that vitamin C degradation is a source of oxoaldehyde stress in vivo in mouse models of aging lens and brain. The same processes were observed whether disruption to normal vitamin C turnover and recycling was generated by additional vitamin C (SVCT2‐transgenic models), or by decreased vitamin C or disease states associated with oxidative stress (SVCT2^±^ knockout, AD, PD).

The concept that vitamin C might damage lenticular proteins during aging was first proposed by Bensch, Fleming, and Lohmann (1985) and has been indirectly supported based on observations from several laboratories including our own (Fan et al., 2010). The closest support was our demonstration that some of the AGEs present in the human lens were elevated in the ASA‐rich lens of the hSVCT2 mouse (Fan et al., 2006). Paradoxically, however, the high levels of MG‐H1 initially found in the human lens (Ahmed et al., 2003), while entirely attributed to glycolysis, were not elevated in the diabetic lens despite elevation of fructose lysine and glucosepane. This major discrepancy is at the heart of the above study.

The lens and the brain share a number of similarities despite vast biological differences. Both lens fibers and neurons are postmitotic cellular systems, both have a high tendency to protein aggregation during aging (Eftekharzadeh, Hyman, & Wegmann, 2016), and both organs are rich in vitamin C that provides crucial antioxidant defense at a young age. Both also suffer from weakening antioxidant homeostasis during aging and impaired GSH levels, which are the hallmark of degenerating neurons and the aging lens nucleus. These shared similarities arguably form the basis for a vicious cycle in which cellular ascorbate‐derived oxoaldehydes accumulate and inflict cellular damage as demonstrated by the accumulation of MG‐H1.

Catalytic metal ions, as suggested by the data, are most likely the major mechanism behind the shift from the antioxidative to the pro‐oxidative role of ASA in vivo. In young and healthy human individuals, iron and copper are strictly sequestered by iron‐ or copper‐binding proteins, that is, transferrin, ferritin (Chen & Paw, 2012), and cuproenzymes (Bhattacharjee, Chakraborty, & Shukla, 2017). Thus, labile iron and copper ions are scarce and ASA oxidation is minimal. However, with age these metal ions accumulate in both human brain and lens (Langford‐Smith et al., 2016; Ramos et al., 2014). Numerous studies have suggested that iron and copper levels in certain brain regions are much higher in neurodegenerative diseases (Bolognin, Messori, & Zatta, 2009). For instance, increased redox‐active iron is seen in the substantia nigra from PD patients compared with healthy individuals (Faucheux et al., 2003), and high copper and iron contents are present in amyloid plaques and neurofilament tangles of Alzheimer's disease (Ward, Zucca, Duyn, Crichton, & Zecca, 2014). In that regard, we have previously demonstrated that CML, which can be formed from ASA itself, can bind redox‐active copper in vivo (Saxena et al., 1999). Yet, while our results are in agreement with the findings of redox‐active metal ions in aging and neurodegeneration, they do not prove that catalytic metals constitute the major pathway to MG‐H1 formation from ascorbate. Similarly, the relative roles ASA oxidation and MG‐H1 play in neurodegeneration remain to be clarified.

**FIGURE 6:**
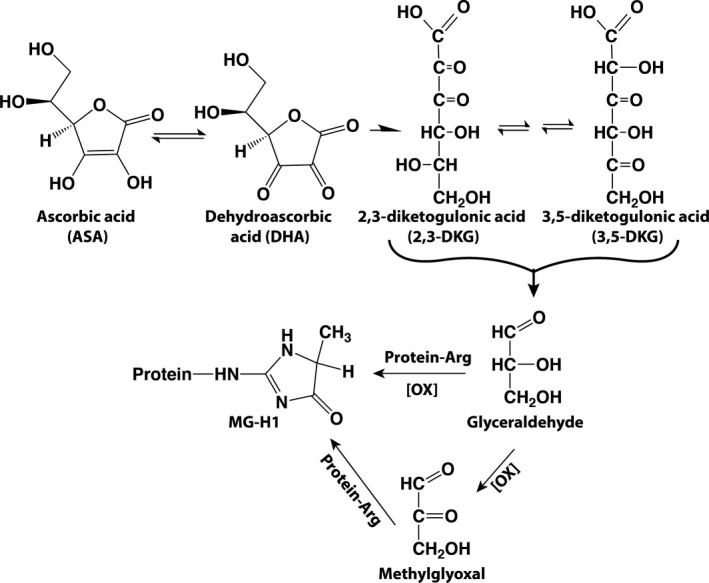
Proposed pathway of ASA oxidation and MG‐H1 inspired by the work of Smuda and Glomb (2013). It should be noted that, in contrast to these authors, we failed to detect glyceraldehyde during in vitro oxidation (Figure 3s), suggesting that ASA‐derived glyceraldehyde became oxidized to methylglyoxal, or presence of a more complex pathway of MG‐H1 formation

The above results lead to several conclusions potentially of translational significance. First, in the presence of impaired redox homeostasis such as in the core of the lens, boosting vitamin C levels could be toxic. One study (Rautiainen, Lindblad, Morgenstern, & Wolk, 2010) in support of such toxicity comes from a cohort study of 25,000 Swedish women in whom vitamin C supplements resulted in increased cataract formation after over 8 years of follow‐up. As to the relationship between Alzheimer's disease (AD), Parkinson's disease (PD), and vitamin C levels, lower levels of plasma vitamin C in AD (Charlton, Rabinowitz, Geffen, & Dhansay, 2004) and PD (Paraskevas et al., 2003) were found. This paradox, however, likely reflects increased oxidative degradation of the vitamin rather than inadequate intake (von Arnim et al., 2012). Indeed in one study, plasma pentosidine levels were elevated in 180 subjects with mild cognitive impairment and 84 AD patients (Monacelli et al., 2015). Similarly, Bar et al. found increased CSF levels of CML in AD patients and elevated pentosidine in plasma from vascular dementia subjects (Bar et al., 2003), both of which can originate from ASA. The profound decrease in GSH level (>40%) in the substantia nigra in PD (Sian et al., 1994) will likely promote ASA oxidation and MG‐H1 formation that is further compounded by impaired GSH‐dependent glyoxalase I scavenging of the methylglyoxal. However, while the above studies using ^13^C‐labeled ASA clearly implicate chemical degradation of ASA as a source of MG‐H1, the precise mechanism of in vivo MG‐H1 formation is elusive. Smuda and Glomb (Smuda & Glomb, 2013) demonstrated that glyceraldehyde is formed at levels ~ 100 times higher than methylglyoxal (Figure 6), a fact we confirmed with the finding of glyceraldehyde in samples of ASA that was incubated for 7 days at 37°C and then remained at 4°C for six months (Fiure S3). While this finding suggests a mechanism as proposed in Figure 6 and is supported by the presence of free glyceraldehyde in vivo (Jonas et al., 1989), the actual mechanism of MG‐H1 formation from ASA could be much more complex.

Looking to the future, while the evidence for impaired ascorbate and GSH homeostasis is already very strong in the lens, stronger data will be needed to implicate vitamin C oxidation products in diseases such as Alzheimer's disease and Parkinson's disease. In that regard, it may be necessary to revisit our older studies on the discovery of pentosidine AGE in the brain from AD patients (Smith et al., 1994) as providing further supportive evidence for ascorbate rather than glucose as the primary source of glycative damage in these diseases.

Finally, it should be stressed that the significance of the above discovery as a culprit of disease is speculative. Most age‐related diseases evolve over many years and are associated with postsynthetic stochastic modifications of macromolecules. Study of the latter provides valuable information as marker of the underlying metabolic stress, be it oxidation, glycation, nitrosylation, ascorbylation, or some other process. However, more precisely implicating the relative contribution of each process to the disease pathology is most often impossible, because pathways are not only highly interconnected, but drugs are rarely able to specifically target the pathway under consideration. Studies are in progress in our laboratory to understand the extent to which loss of a positive charge due to MG‐H1 formation in lens crystallins contributes to crystallin precipitation in age‐related cataract.

## 4 EXPERIMENTAL PROCEDURES

### 4.1 Animals

All animal experiments were conducted in accordance with procedures approved by the Case Western Reserve University Animal Care Committee and conformed to the ARVO Statement for Use of Animals in Ophthalmic and Vision Research.

### 4.2 Human‐derived samples

All experiments using human‐derived samples were approved by the Institutional Review Board (IRB) of Case Western Reserve University and Augusta University, and abide by the Declaration of Helsinki principles.

### 4.3 Human brain samples

Frozen tissue samples of normal human brain neocortex (*n* = 30; ages: 31–95, postmortem interval: 4–27 hr, average = 16.3 hr) pathologically confirmed AD cases hippocampus (*n* = 16; ages: 59–91, average = 79.75 years; postmortem interval: 2–9 hr, average = 5.1 hr) and age‐matched normal control hippocampus (*n* = 15; ages: 67–86, average = 75.5 years, postmortem interval: 2–22 hr, average = 13.25 hr) were collected for MG‐H1 analysis by LC/MS. Six Parkinson's disease, six age‐matched normal control, and four young normal control midbrain paraffin‐embedded tissue samples were kindly provided by Dr. Jiri Safar from the Department of Pathology at University Hospitals of Cleveland. The ages of PD subjects were 74 year, 78 year, 88 year, 79 year, 83 year, and 78 year; the ages of age‐matched control subjects were 55 year, 71 year, 60 year, 77 year, 77 year, and 83 year; and the ages of young control subjects were 13 year, 1 year, 1 year, and 9 year. Six‐micrometer‐thickness sections were achieved for immunohistochemistry stain.

### 4.4 Human and mouse lens samples

Human lenses were from the National Disease Research Interchange (NDRI) (Philadelphia, PA). Categorization was made by gender and race, and the primary cause of death was based on information provided by NDRI. All lenses were classified according to the degree of pigmentation into Pirie grade I (*n* = 20), grade II (*n* = 16), grade III (*n* = 9), and grade IV (*n* = 0) as in the past. Mouse lenses were collected as previously described (Fan et al., 2006). All the lenses were stored at −80°C until use.

### 4.5 Statistical methods

All values were expressed as means ± *SD*. Statistical analysis was performed according to methods previously described in detail (Sell, Kleinman, & Monnier, 2000). In brief, regression analysis, Student's *t* test, one‐way ANOVA, Spearman's correlations, and the Mann–Whitney test were computed using SPSS software. Testing for homogeneity of variance was done using either the *F* test or the Burr–Foster Q test, as previously described (Sell et al., 2000). Linear regression analysis, including computation of regression line and its 95% confidence intervals (CI) of prediction, was done using SigmaPlot 13.0 software (Systat Software, Inc., San Jose, CA). Data were transformed with either the square‐root or log *y* transformations. Significance was considered *p* < .05.

### 4.6 Additional methods

Sample processing, mass spectrometry analysis, and immunohistochemistry methods are provided in supplemental materials.

## CONFLICT OF INTEREST

The authors declare no conflict of interest.

## AUTHOR CONTRIBUTIONS

XF and VMM conceived the research; XF, DS, CH, BW, SL, and DWW acquired the data; SS, XZ, TJK, JS, and FEH contributed critical reagents; XF and VMM supervised the research; XF, BW, and VMM analyzed and interpreted the data; XF and VMM wrote the manuscript, FEH provided assistance with editing.

## Supporting information

Fig S1‐S3

## Data Availability

The authors will provide detailed description of methods and original data upon request.
