# Supplementary material for: Vitamin C is a source of oxoaldehyde and glycative stress in age‐related cataract and neurodegenerative diseases
Source: Aging Cell. 2020 Jun 21;19(7):e13176. doi: 10.1111/acel.13176 (PMC7418511; doi:10.1111/acel.13176)
Supplement: Supplementary file 1 — Fig S1‐S3 [file ACEL-19-e13176-s001.pdf]

SUPPLEMENTAL MATERIALS AND METHODS

Reagents

All chemicals used were of analytical reagent grade. Milli-Q water was used for the preparation of standards and reagents. $C_{1,2}$-C^{13}-ASA, C_3-C^{13}-ASA, C_5-C^{13}-ASA, and U6-C^{13}-ASA were purchased from Omicron Biochemical, Inc. (South Bend, IN). N-lauroylsarcosine sodium salt and all other chemical reagents were from Sigma-Aldrich (St. Louis, MO).

Animals

Animals were housed under diurnal lighting condition and allowed free access to food and water. The lens specific human Sodium dependent Vitamin C Transporter 2 (hSVCT2) transgenic mice in C57BL6 background was generated by us as previously described(Fan et al., 2006). Brain tissue from systemic SVCT2 bacterial artificial chromosome (BAC) transgenic mice (BAC) (Harrison et al., 2012), and SVCT2$^{+/-}$ heterozygous knockout mice(Harrison et al., 2010) was provided by Dr. Fiona E. Harrison, Vanderbilt University Medical Center. The systemic gamma glutamyl-cysteine ligase modifier subunit knockout mice (Gclm$^{-/-}$)(Cole et al., 2011) was provided by Terrence J. Kavanagh, University of Washington.

Preparation of lens protein extract

Human lenses were decapsulated and homogenized in 5mM Chelex treated sodium phosphate buffer (pH 7.4) with protease inhibitors added (Roche, IN) (2.0ml per lens) in a Con-Torqueglass homogenizer (Eberbach) for 3min on ice. The homogenate was centrifuged at 20,000 x g for 30min at 4°C and separated into supernatant and pellet. The pellet was suspended in 2.0ml of ice-cold buffer and re-centrifuged. The two supernatants were pooled and dialyzed against 5mM Chelex treated sodium phosphate buffer (pH 7.4) for 48hrs with buffer change every 24hours at 4°C. The protein concentration was measured by BCA protein assay (Thermo Scientific, Rockford, IL).

Preparation of human brain insoluble protein extract

Insoluble proteins from brains were extracted with the use of sarkosyl following a previously described method (Goedert, Spillantini, Jakes, Rutherford, & Crowther, 1989; Ittner et al., 2008). Briefly, human brain hippocampus or frontal cortex tissues (5mg wet weight/ ml) were homogenized in 100 mM MES (pH 6.8), 750 mM NaCl, 1mM dithiothreitol and protease inhibitors (P8340, Millipore Sigma, St. Louis, MO). Homogenates were incubated for 20 min at

4°C and centrifuged at 11,000×g for 20 min at 4°C. The supernatant was centrifuged at 100,000×g for 60 min at 4°C. For the isolation of insoluble proteins, the pellets were twice extracted in 1:10 (w/v) extraction buffer (10 mM Tris (pH 7.4), 10% sucrose, 850 mM NaCl, 1 mM EGTA) and centrifuged at 15'000×g for 20 min at 4°C. The supernatants were combined, and sarkosyl was added to a final concentration of 1%, followed by 1 hour of incubation at room temperature on a head-over-head rotor. The samples were centrifuged at 100,000×g for 45 min at 4°C to pellet the sarkosyl-insoluble proteins. The pellet was washed 3x with Milli-Q water and subjected to acid hydrolysis as described above.

In vitro incubation using human brain hippocampal tissue

5 AD and 5 healthy control hippocampal tissues were used in the *in vitro* incubation. Each sample was divided into 4 groups, and 5mg wet weight of tissue was used in each group. For group A and B, tissues were homogenized in 250µl 50mM sodium phosphate buffer, pH 7.4, 5mM DTPA. The homogenate mix was dialyzed against 2L 50mM sodium phosphate buffer, pH 7.4, 5mM DTPA for 48hr with change of dialysis buffer every 24hr. The homogenate mix was then dialyzed against Chelex-treated 50mM sodium phosphate, pH7.4 for additional 48hr and change of dialysis buffer every 24hr. For group C and D, tissues were homogenized in 250µl Chelex-treated 50mM sodium phosphate buffer, pH7.4. 5mM final concentration of ascorbic acid was added to group B and D. All four groups were incubated for 48hr at 37°C. The proteins were precipitated with an equal volume of pre-cold 20% trichloroacetic acid (TFA) and keep on ice for 30min. The pellet was washed three times by 500µl/each ethyl ether for delipidation and MG-H1 analysis by LC/MS as described below.

In vitro incubation of proteins with glycating agents

Lens protein extract (20mg/ml) in 50mM sodium phosphate buffer, pH7.4 from 4 months old human lens was prepared as described above and incubated with 3mM ASA in 5mM Chelex treated 50 mM potassium phosphate buffer (pH 7.4) under either anaerobic or aerobic condition for 7 days at 37°C in the dark. For other glycation experiments lens protein extract (20mg/ml) from 4 months old human or bovine serum albumin (BSA) (Sigma-Aldrich, Catalog No. A9306) was incubated with 3mM ASA, 10nM-10µM MGO, or 10nM-10µM glyceraldehyde in anaerobic condition for 7 days at 37°C in the dark. The protein was then dialyzed against phosphate buffer

saline (PBS) for 24 hours at 4ºC and twice against water for 48 hours at 4ºC and lyophilized. Half of the incubated protein (~2.5mg) was used for acid hydrolysis, as previously described (Fan et al., 2006). The other half of the protein was used for western-blot analysis.

For ^{13}C-labeled ASA incubation, protein (20mg/ml) from 4 months old human lens extract was incubated with 3mM $C_{1,2}$-C^{13}-ASA, C_3-C^{13}-ASA or C_5-C^{13}-ASA in 5mM Chelex treated potassium phosphate buffer (pH 7.4) for 7 days in anaerobic condition at 37ºC in the dark. At the end of incubation, the proteins were dialyzed with 3K Slide-A-Lyzer dialysis cassette (Thermo Scientific, Rockford, IL), and concentrated with 3K Centricon (Millipore, Darmstadt, Germany). The proteins were reconstituted in 50mM ammonium bicarbonate and 6M-urea buffer for Tandem mass analysis.

Preparation of brain tissue acid hydrolysate

3mg wet weight of brain samples was extracted for 24 hours at 4°C with 2:1 chloroform: methanol in glass tubes followed by washing with cold methanol and then water.

The delipidated brain tissue samples were placed in a 13 × 100–mm glass tube with a Teflon-lined screw cap(Sell, Carlson, & Monnier, 1993). Each sample was acid hydrolyzed for 18 h with 3 ml of 6 M HCl as described(Fan et al., 2009). To minimize discoloration and artifact formation during acid hydrolysis, the concentration of tissue to acid was maintained at ∼1 mg/ml. In addition, the acid was degassed under vacuum followed by purging with argon by bubbling for at least 20 min. The acid was immediately pipetted into each tube which was thoroughly purged the tube with argon before sealing with the screw cap. The samples were dried by evaporating the acid with a SpeedVac Concentrator (Savant; ThermoFisher) and reconstituted with 300μL water and filtered using a Spin-X centrifuge tube filter (Costar; Corning Inc., Corning, NY). The amino acids content of each sample was determined by the ninhydrin assay(Fan et al., 2009).

Methylglyoxal Assay

Methylglyoxal levels were determined by o-phenylenediamine (*o*-PD) derivatization method, as described(Chaplen, Fahl, & Cameron, 1996). In brief, the lens extract was homogenized in 100μl ice-cold 5M perchloric acid (PCA) and centrifuged, and the supernatant was passed through C18-SPE cartridge (Waters, Milford, MA) before derivatization with *o*-PD. The quinoxaline derivative

of methylglyoxal (2-methylquinoxaline (2-MQ) was separated and quantitatively determined by HPLC using 5-methylquinoxaline (5-MQ) as the internal standard.

Sample Digestion and LC/MS Analysis

Samples were dissolved in 8M urea with 50 mM ammonia bicarbonate, reduced with 10 mM dithiothreitol (DTT) at 25°C for 1 h. The free cysteine was alkylated with iodoacetamide at a final concentration of 55 mM in 50 mM ammonium bicarbonate for 30 min in the dark. The solution was 8 times diluted and digested with mass spectrometry grade Lys-C (Wako, Japan) for 2 h following trypsin (Promega, Madison, WI) digestion overnight.

The digests were analyzed with LC-MS/MS using Orbitrap Elite Hybrid Mass Spectrometer (Thermo Electron, San Jose, CA) coupled with a Waters nanoAcquity UPLC system (Waters, Taunton, MA). A full scan was obtained in the Orbitrap at 120,000 resolution followed by 20 MS/MS scans with collision-induced dissociation of the peptide ions at a normalized collision energy of 35% during 90 min total analysis time. Obtained data were submitted to database search through Mascot search engine (version 2.4.0, Matrix Science) against SwissProt human database (20266 sequences), with the setting of 10 ppm for parent ions and 0.8 Da for-product ions. Maximum three missed cleavages were allowed. Carbamidomethylation of Cys, oxidation of Met, and various ascorbylation endpoints were set as variable modifications. No fixed modification setting was made. Residue ascorbylation included modifications at arginine residues with MG-H1 (mass shift of 54.0106 Da) and CEL (mass shift of 72.0211 Da), as well as their corresponding ascorbylations with one or two ^{13}C labeled MG-H1 and CEL. Significance threshold value was $p<0.05$. The ascorbylation sites were further verified through each tandem mass spectrum of the modified peptides by manual examination. The mass spectrometry proteomics data have been deposited to the ProteomeXchange Consortium via the PRIDE partner repository with the dataset identifier PXD016796.

Determination of MG-H1 with LC-MS

Levels of methylglyoxal hydroimidazolone isomer 1 (MG-H1) were determined by electrospray positive ionization–mass spectrometric multiple reaction monitoring (ESI + MRM) as described(Fan et al., 2010). The liquid chromatography-tandem mass spectrometry system consisted of a Waters Alliance 2695 Separations Module HPLC coupled to a Micromass Quattro

Ultima triple quadropole mass spectrometer, both operated with a computer using MassLynx Software V4.1 (Waters Corp., Milford, MA). The transition (*m/z* molecular ion→*m/z* product ion) of MG-H1 (229→114) was monitored by MRM program.

Stereotaxic injections of C^{13}-labeled ascorbic acid

Following induction in Isoflurane anesthetic (3.0-3.5%, 1L/min O2), mice were mounted into a stereotaxic frame and anesthetic state maintained under Isoflurane. Body temperature was maintained at 38°C with a heating pad. Upon confirmation of anesthesia, the head was shaved, cleaned with betadine and 70% EtOH, and an injection of marcaine (S.C.) was administered within the scalp. A smidline incision was made from ~5mm posterior of the nose along the midline to ~3mm posterior of lambda. Two holes (~0.7mm diameter) were drilled bilaterally above each lateral ventricle. A 5µL NeuroS Hamilton syringe (33 gauge needle, Hamilton Company) loaded with 20mM C^{13}-labeled ascorbic acid in 100mM potassium phosphate buffer (pH 7.4) was lowered into a single ventricle and 10 µL of solution infused at a rate of 100nL/min. Following the first injection, the syringe was raised out of the brain, and the process was repeated on the contralateral side. After completion of the second injection, the craniotomies were sealed with dental wax, and the wound margin closed with Vetbond (3M). Rimadyl (Carprofen, 5mg/kg, s.c., Pfizer animal health) was administered daily for 3 days post-op. 6 mice in each group were used for this study.

Immunohistochemical analyses

For immunohistochemical analysis, 6 µm paraffin-embedded sections from the midbrains of six PD human cases, 6 age-matched control and 4 young healthy individuals were stained with the primary antibodies directed against MG-H1 (1:500, Gift from Dr. Michael Brownlee, Albert Einstein College of Medicine, NY). Slides were immunostained simultaneously under identical conditions, using the Polink-2 HRP DAB section system (Golden Bridge International, Inc., Mukilteo, WA) following the instructions from the manufacturer. The staining intensity was measured via ImageJ software as described (Jensen, 2013). The image was first converted to RGB stack and adjust to montage, the same minimal and maximal thresholds were set for entire images. The calculated area was reported as relative staining intensity. The average of eight images in each immunohistochemically stained sample were calculated for statistical analysis.

Western Blot

Human and mouse lens protein extract was prepared in 50mM potassium phosphate buffer (pH 7.4), homogenized, and centrifuged. Whole protein extract was further processed for immunoblot analysis and probed for MG-H1 modification using anti-MG-H1 monoclonal antibody (1:2000)(Kilhovd et al., 2003). All data were normalized to the total level of protein stained by Ponceau S.

NMR Spectroscopy

Ascorbic acid and D-glyceraldehyde were purchased from Sigma as ultragrade chemicals (Ascorbic acid, A5960; D-glyceraldehyde G-5001) while methylglyoxal solution was purchased as a 40% solution in water (Sigma M0252) and distilled in our laboratory before use. Phosphate buffered saline (PBS) was prepared by dissolving a PBS tablet (Sigma P4417) in D_2O before the addition of either ascorbic acid, glyceraldehyde or the distilled methylglyoxal solution. Totals ~600 µl of each solution contained in D_2O were placed in NMR tubes and subjected to NMR analysis by an 800 MHz NMR Bruker instrument (North East Ohio High Field NMR Facility, Case Western Reserve University, Cleveland, Ohio). Each analysis was conducted for at least 1.6 hrs ~ 4096 scans.

SUPPLEMENTAL FIGURES:

Figure S1

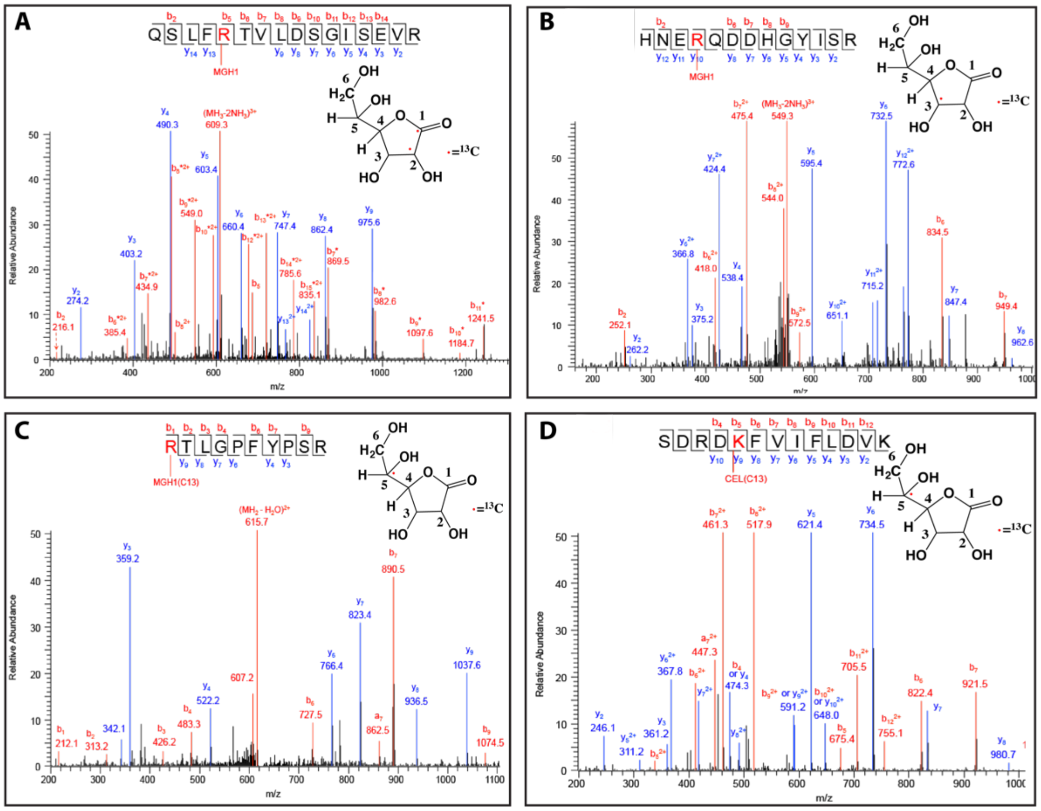

Figure S1. Representative tryptic peptide mass spectra from human lens extract modified by incubation under aerobic conditions for one week at 37°C with ^{13}C labeled ASA at carbon positions 1 and 2, 3 or 5. (A) $C_{1,2}$-^{13}C-ASA modification only produced ^{12}C-MG-H1. (B) C_3-^{13}C-ASA modification only produced ^{12}C-MG-H1 (C) C_5-^{13}C-ASA modification only produced ^{13}C-MG-H1. (D) C_5-^{13}C-ASA modification only produced ^{13}C-CEL. (E) Structural relationship between ascorbic acid and MG-H1 formation showing carbons C4, C5 and C6 in red color being incorporated into MG-H1 and CEL structures.

Figure S2

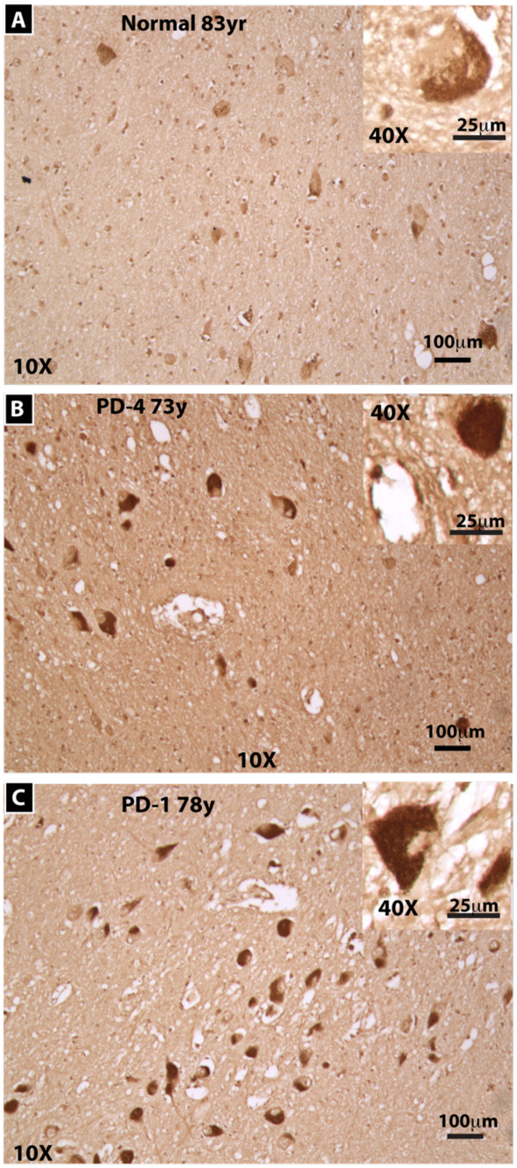

Figure S2. Other representative immunohistochemistry of MG-H1 in PD substantia nigra compared to young and age-matched control midbrain. (A) 83 year old normal brain; (B) 73 year old PD brain; (C) 78 year old PD and brain. The main images are taken at 10x magnification and the insets in upper right are 40x.

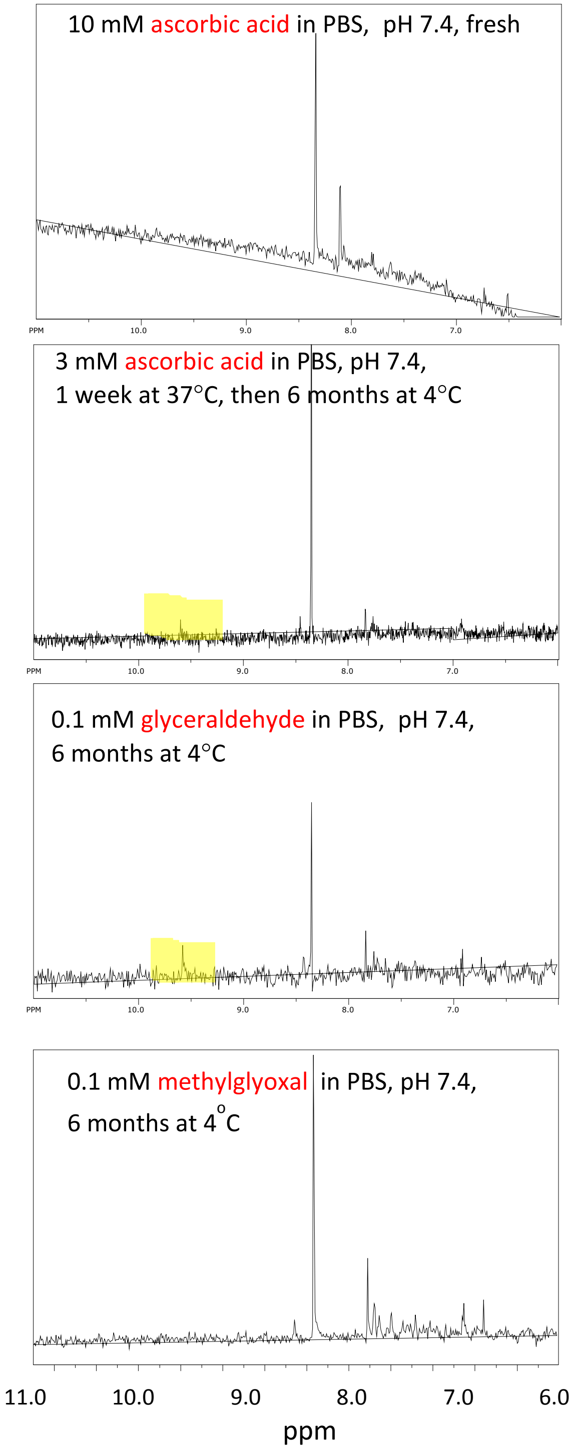

Figure S3: Upfield 1H-NMR spectroscopy (from 6 to 11 ppm) of aqueous solutions of ascorbic acid, D-glyceraldehyde and methylglyoxal stored in phosphate buffered saline (PBS) up to six months in the refrigerator. Shown in yellow is the presence of the alpha proton of D-glyceraldehyde at 9.6. ppm in 3 mM ascorbic acid that was first incubated at 37°C for 1 week, and then 6 months at 4°C. Note that the signal is absent in fresh 10 mM ascorbic acid and 0.1 mM distilled methylglyoxal. For a reference spectrum for glyceraldehyde see http://www.bmrb.wisc.edu/metabolomics/mol_summary/show_data.php?id=bmse000298&whichTab=1

REFERENCE

Chaplen, F. W., Fahl, W. E., & Cameron, D. C. (1996). Method for determination of free intracellular and extracellular methylglyoxal in animal cells grown in culture. *Anal Biochem, 238*(2), 171-178. doi:10.1006/abio.1996.0271

Cole, T. B., Giordano, G., Co, A. L., Mohar, I., Kavanagh, T. J., & Costa, L. G. (2011). Behavioral Characterization of GCLM-Knockout Mice, a Model for Enhanced Susceptibility to Oxidative Stress. *J Toxicol, 2011*, 157687. doi:10.1155/2011/157687

Fan, X., Reneker, L. W., Obrenovich, M. E., Strauch, C., Cheng, R., Jarvis, S. M., . . . Monnier, V. M. (2006). Vitamin C mediates chemical aging of lens crystallins by the Maillard reaction in a humanized mouse model. *Proc Natl Acad Sci U S A, 103*(45), 16912-16917. doi:10.1073/pnas.0605101103

Fan, X., Sell, D. R., Zhang, J., Nemet, I., Theves, M., Lu, J., . . . Monnier, V. M. (2010). Anaerobic vs aerobic pathways of carbonyl and oxidant stress in human lens and skin during aging and in diabetes: A comparative analysis. *Free Radic Biol Med, 49*(5), 847-856. doi:S0891-5849(10)00355-2 [pii]

10.1016/j.freeradbiomed.2010.06.003

Fan, X., Zhang, J., Theves, M., Strauch, C., Nemet, I., Liu, X., . . . Monnier, V. M. (2009). Mechanism of lysine oxidation in human lens crystallins during aging and in diabetes. *J Biol Chem, 284*(50), 34618-34627. doi:M109.032094 [pii]

10.1074/jbc.M109.032094

Goedert, M., Spillantini, M. G., Jakes, R., Rutherford, D., & Crowther, R. A. (1989). Multiple isoforms of human microtubule-associated protein tau: sequences and localization in neurofibrillary tangles of Alzheimer's disease. *Neuron, 3*(4), 519-526.

Harrison, F. E., Best, J. L., Meredith, M. E., Gamlin, C. R., Borza, D. B., & May, J. M. (2012). Increased expression of SVCT2 in a new mouse model raises ascorbic acid in tissues and protects against paraquat-induced oxidative damage in lung. *PLoS One, 7*(4), e35623. doi:10.1371/journal.pone.0035623

Harrison, F. E., Dawes, S. M., Meredith, M. E., Babaev, V. R., Li, L., & May, J. M. (2010). Low vitamin C and increased oxidative stress and cell death in mice that lack the sodium-dependent vitamin C transporter SVCT2. *Free Radic Biol Med, 49*(5), 821-829. doi:10.1016/j.freeradbiomed.2010.06.008

Ittner, L. M., Fath, T., Ke, Y. D., Bi, M., van Eersel, J., Li, K. M., . . . Gotz, J. (2008). Parkinsonism and impaired axonal transport in a mouse model of frontotemporal dementia. *Proc Natl Acad Sci U S A, 105*(41), 15997-16002. doi:10.1073/pnas.0808084105

Jensen, E. C. (2013). Quantitative Analysis of Histological

Staining and Fluorescence Using

ImageJ. *THE ANATOMICAL RECORD, 296*, 378-381.

Kilhovd, B. K., Giardino, I., Torjesen, P. A., Birkeland, K. I., Berg, T. J., Thornalley, P. J., . . . Hanssen, K. F. (2003). Increased serum levels of the specific AGE-compound methylglyoxal-derived hydroimidazolone in patients with type 2 diabetes. *Metabolism, 52*(2), 163-167. doi:10.1053/meta.2003.50035

Sell, D. R., Carlson, E. C., & Monnier, V. M. (1993). Differential effects of type 2 (non-insulin-dependent) diabetes mellitus on pentosidine formation in skin and glomerular basement membrane. *Diabetologia, 36*(10), 936-941.
